# Synergistic Fluoride Adsorption by Composite Adsorbents Synthesized From Different Types of Materials—A Review

**DOI:** 10.3389/fchem.2022.900660

**Published:** 2022-05-04

**Authors:** Yifei Wei, Li Wang, Hanbing Li, Wei Yan, Jiangtao Feng

**Affiliations:** Xi’an Key Laboratory of Solid Waste Recycling and Resource Recovery, Department of Environmental Science and Engineering, School of Energy and Power Engineering, Xi’an Jiaotong University, Xi’an, China

**Keywords:** fluoride adsorption, metal oxides/hydroxides, carbon-based adsorbents, biopolymer, modification

## Abstract

The reduction of fluoride concentrations in water is one of many concerns. Adsorption is the most widely used technology for fluoride removal and the center to development of adsorption technology is the improvement of adsorbents. This review classifies the typical fluoride removal adsorbents into four types: metal oxides/hydroxides, biopolymers, carbon-based, and other adsorbents. The exploitation of new materials and the synthesis of composite materials are two ways of developing new adsorbents. In comparison to the discovery of novel adsorbents for fluoride adsorption, research into the composite synthesis of different types of conventional adsorbents has proliferated in recent years. The traditional adsorbents used the earliest, metal oxides, can act as active centers in a wide range of applications for modifying and compounding with other types of adsorbents. This study emphasizes reviewing the research on fluoride removal by composite adsorbents synthesized from different types of metal-modified materials. Seven factors were compared in terms of material characterization, initial fluoride concentration, adsorbent dose, pH, temperature, reaction time, and maximum adsorption capacity. The modification of composite adsorbents is facile and the synergistic effect of the different types of adsorbents significantly improves fluoride adsorption capacity. Metal composite adsorbents are synthesized by facile coprecipitation, hydrothermal, or impregnation modification methods. The adsorption mechanisms involve electrostatic attraction, ion exchange, complexation, and hydrogen bonding. The fluoride adsorption capacity of composite adsorbents has generally improved, indicating that most modifications are successful and have application prospects. However, to achieve significant breakthroughs in practical applications, numerous issues such as cost, separation/regeneration performance, and safety still need to be considered.

## 1 Introduction

Fluoride ions in water have a strong affinity with positively charged elements such as calcium (Bhatnagar et al., 2011), which is a major component of human bone and tooth structure (Rehman et al., 2015). Low concentrations of fluoride in drinking water (0.5–1.5 mg/L) (Chen et al., 2017) can strengthen bones and prevent dental caries, while excessive concentrations of fluoride (4–10 mg/L) can cause diseases such as fluorosis, osteoporosis, brittle bones, brain damage, and several thyroid disorders (Kumar et al., 2020). Excessive fluoride concentrations in water have become a public health concern in developing countries. High concentration (>10 mg/L) fluorinated wastewater is easier to treat and can be removed or reduced by coagulation, precipitation, electrochemistry, and other methods. The treatment of low concentration (2–10 mg/L) fluorinated wastewater is relatively difficult and is also a current research hotspot. The treatment methods include adsorption (Lin et al., 2016), membrane separation, ion exchange (Xu et al., 2017), nanofiltration (Wang A. et al., 2018), reverse osmosis (Ye et al., 2019), and electrodialysis (Fan et al., 2019). Among them, the adsorption method has the advantages of low cost, high flexibility, simple operation, and high efficiency (Sarkar et al., 2019). It is the most widely used and the treatment effect is more satisfactory.

The treatment effectiveness of the adsorption method is influenced by a number of factors, including adsorbent properties, fluoride ion selectivity, compatibility, solution pH, temperature, co-existing ions, and contact time (Pigatto et al., 2020). It mainly depends on the adsorbent properties such as particle size, pore size structure, zero charge point (*pH*_*PZC*_), and specific surface area (*S*_*BET*_) (Biswas et al., 2017). High specific surface area (developed pore structure) and ideal chemical surface (abundant functional groups) are two essentials for effective removal of fluoride by adsorbents. Although not systematically categorized, this review found that the main traditional sorbents frequently used for fluoride removal are metal oxides/hydroxides (Dhillon et al., 2017), low-cost carbon materials (Zhang X. et al., 2021), biomolecular materials (Jia et al., 2018), and others such as clay, hydroxyapatite, and graphite. This review classifies the more researched fluoride removal adsorbents into four categories: metal oxide/hydroxide adsorbents, biopolymer adsorbents, carbon-based adsorbents, and other adsorbents (industrial waste, minerals, etc.).

For the development of new adsorbents, the discovery of novel adsorbents that have never been used before and the composite material synthesis by combining traditional adsorbents are the two main approaches to improve adsorption capacity. In comparison to the discovery of novel adsorbents that have never been used before, research into the composite synthesis of different types of conventional adsorbents for fluoride adsorption has proliferated in recent years. The emphasis of this study is placed on the fluoride adsorption effect of this adsorbent compounded from different types of conventional adsorbents. The study of other types of metal-modified adsorbents accounts for a major part. A total of seven factors were compared in terms of material characterization, initial fluoride concentration, adsorbent dose, pH, temperature, reaction time, and maximum adsorption capacity.

## 2 Conventional Types of Adsorbents

### 2.1 Metal Oxide/Hydroxide Adsorbents

Metal oxide/hydroxide nanoparticles were reported to show an affinity for fluoride and high performance in fluoride removal. The high reactivity (Lanas et al., 2016), specificity, specific surface area (Rathore and Mondal 2017), stability, and self-assembly potential have attracted attention in fluoride removal studies. Nanoscale dimensions with desirable physicochemical properties, such as high density of hydroxyl ions on the high specific surface area, will further enhance the fluoride adsorption capacity.

#### 2.1.1 Aluminum Oxide/Hydroxide

**FIGURE 1:**
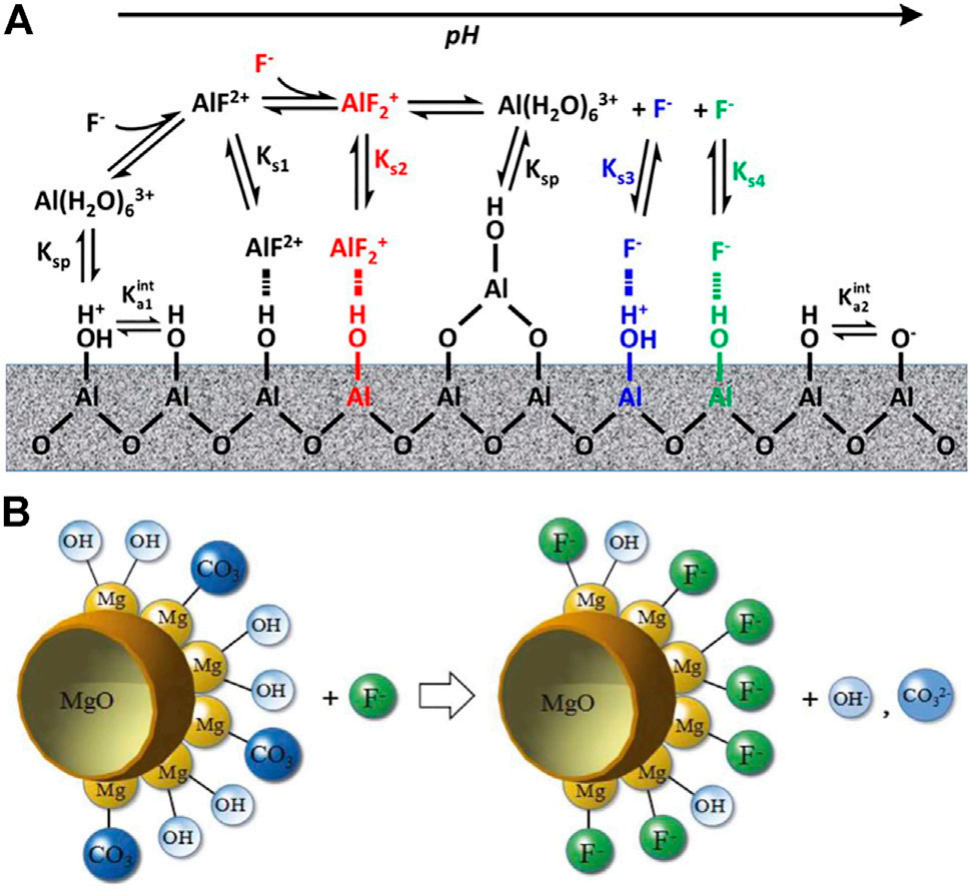
Mechanism of fluoride adsorption by activated alumina **(A)** (Lin et al., 2020) and MgO **(B)** (Zhang Y. et al., 2021).

Aluminum oxide/hydroxide was the earliest studied and used adsorbents for fluoride removal (Chinnakoti et al., 2016a). Typically, aluminum hydroxide is first prepared by electrolysis or pyrolysis and then partially converted to aluminum oxide by calcination. One of the advantages of aluminum oxide/hydroxide adsorbents is the large specific surface area (Hafshejani et al., 2017), as shown in Table 1, and in general, *S*_*BET*_ > 200 m^2^/g. Generally, high *pH*_*PZC*_ allows its surface to appear positively charged in water (Dhawane et al., 2018). Several studies have reported that the mechanism of fluoride adsorption by alumina mainly consists of electrostatic attraction and ion exchange (Rathore and Mondal 2017), as shown in Figure 1A; the monodentate complex Al-F is the major formation after adsorption (Kang et al., 2018; Lin et al., 2020). Kang et al. (2018) synthesized an amorphous alumina microsphere using solvothermal reaction and calcination, with *S*_*BET*_ = 400 m^2^/g and a maximum adsorption capacity of 129.4 mg/g; they proposed that the adsorption mechanism involves chemical reaction and pore filling in addition to ion exchange and electrostatic attraction. However, aluminum is easily leached out in aqueous solutions, especially under acidic conditions (Lin et al., 2020), leading to high concentration of aluminum residues in drinking water, which is also a major threat to human health.

**TABLE 1:** Summary of the preparation methods, characteristics, and adsorption mechanisms of four traditional adsorbents.

Adsorbents	Preparation method	Dimension	*S*_*BET*_ (m^2^/G)	Aperture (nm)	*pH*_*PZC*_	Adsorption mechanism	Ref
Cactus-like amorphous alumina oxide microspheres	Solvothermal method without templates	40 μm	419.6	5.3	6.6	Chemical coordination, electrostatic attraction, and ion exchange	Kang et al. (2018)
Nano γ-alumina	Surfactant-assisted combustion	—	221	—	6.5	—	Chinnakoti et al. (2016a)
Mesoporous micro alumina	γ-AlOOH calcined at 873 K	0.9 mm	254.1	12.96	9.0	Electrostatic attraction	Lanas et al. (2016)
Al_2_O_3_ nanoparticles	Flame spray pyrolysis (FSP)	9.8 nm	213	93	—	Electrostatic attraction	Hafshejani et al. (2017)
Porous-layered Al_2_O_3_	Roasting of AlFu MOFs	—	329.3	3.8	—	Ion exchange, complexation	Yang et al. (2020)
Activated alumina	Al_2_O_3_ cauterized at 673 K	1–3 mm	185.6	5.1	8.5	Lewis acid and base	Dhawane et al. (2018)
Aluminum oxide/hydroxide	Electrolysis, calcination at 973 K	1.5 mm	253.2	4.7	7.52	Electrostatic attraction	Rathore and Mondal (2017)
Cubical ceria nano-adsorbent	Coprecipitation, calcination at 473 K	4.5 nm	98	2.62	6	Ligand exchange, complexation	Dhillon et al. (2016)
CeO_2_ nanorods	Hydrothermal at 373 K	20*200 nm	111.4	8.65	—	Ce^3+^-O defect, ion exchange,	—
Pore filling	Kang et al. (2017)	—	—	—	—	—	—
CeO_2_ octahedron	Hydrothermal at 453 K	14 nm	160.2	9.66	—	—	—
CeO_2_ nanocubes	Hydrothermal at 473 K	25 nm	55.8	15.1	—	—	—
CeCO_3_OH nanosphere	Hydrothermal	250 nm	10.6	15.5	—	Electrostatic attraction, ion exchange	Zhang et al. (2016b)
Porous MgO nanoplates	Solvothermal, calcination	—	47.4	3.3	—	Ligand exchange	Jin et al. (2016)
Hollow MgO spheres	Hydrothermal, calcination at 773 K	2 μm	—	—	10	Ligand exchange	Zhang et al. (2021b)
Microsphere-like MgO	Hydrothermal, calcination at 773 K	46 μm	120.7	5.12	-	Ion exchange	Lee et al. (2017)
Pillar-like MgO	Hydrothermal, calcination at 773 K	2*20 μm	99.44	6.26	-	Ion exchange	Lee et al. (2017)
γ-Fe_2_O_3_ nanoparticles	Precipitation	5–20 nm	—	—	8.13	Complexation	Jayarathna et al. (2015)
Trititanate nanotubes	Hydrothermal at 403 K, 1 h	8–12 nm	282	-	2.5	Ion exchange, electrostatic attraction	Chinnakoti et al. (2016b)
TiO_2_	Solvothermal method	1 μm	31.9	—	6.5	Complexation	Zhou et al. (2019b)
Lanthanum alginate bead	LaCl_3_ cross-linking	1 mm	2.618	1.441	—	Ion exchange	Huo et al. (2011)
Biopolymer pectinandalginate	Glutaraldehyde cross-linking mixture	—	—	—	—	—	Raghav et al. (2019)
Porous zirconium alginate	CaCl_2_ cross-linking SA, Zr(NO_3_)_4_ immersion	2 mm	3	—	—	Electrostatic attraction, ion exchange	Qiusheng et al. (2015)
Shell biochar	Calcination at 1073 K	0.5 mm	4	413	6	Complexation	Lee et al. (2021)
Nanoscale rice husk biochar	Calcination at 873 K, ball milling	—	—	—	—	Ion exchange	Goswami and Kumar (2018)
Mustard ash biochar	Carbonization at 873 K	—	—	—	—	—	Jadhav and Jadhav (2021)
Peanut shell biochar	Pyrolysis at 673 K, 1 h	—	98	7.05	—	—	Kumar et al. (2020)
Rhodophyta biochar	Calcined in muffle for 2 h	75 μm	320	1.28	5.4	Complexation	Naga Babu et al. (2020)
Rice husk biochar	Pyrolysis at 698 K in tube furnace	—	3	13.29	5.9	Ion exchange	Yadav and Jagadevan (2020)
Activated sugarcane ash	Burning at 773 K in muffle furnace	150 μm	64	—	—	Ion exchange	Mondal et al. (2016)
KOH-treated jamun seed	KOH activation, pyrolysis at 1173 K	—	748	2.19	4.9	Ligand exchange	Araga et al. (2017)
KOH-treated activated carbon	Carbonization with solid KOH at 873 K	—	1,006	1.95	6.11	Protonation, ion exchange	Bhomick et al. (2019)
Activated carbon	Surfactant modification	—	—	—	6.86	Electrostatic attraction	Chen et al. (2019)
Coconut-shell carbon	Carbonization at 1173 K in tube furnace	500 nm	358	—	—	Electrostatic attraction	Araga et al. (2019)
Chicken bone biochar	Burning at 873 K in muffle furnace	159 μm	126	—	—	Ion exchange	Herath et al. (2018)
Bone char	—	0.8 mm	104	11.4	8.4	Electrostatic attraction	Medellin-Castillo et al. (2014)
Bovine bone biochar	Burning at 773 K in muffle furnace	—	115	3.823	2.2	Ion exchange	Zhou et al. (2019a)
Kaolinite	Alkali–hydrothermal	—	18	4	5	Ion exchange	Wang et al. (2017b)
Activated clay	Sulfuric acid activation	—	167	4.9	—	—	Guiza et al. (2019)
Fly ash–paper mill lime mud	Mixing, calcination	60 μm	58.9	—	—	Ligand exchange, complexation	Ye et al. (2019)
Natural clay	—	-	-	—	8	—	Nabbou et al. (2019)
Natural pumice	—	200 μm	9.5	—	3	—	Dehghani et al. (2016)
Natural zeolite	NaOH activation	—	—	—	—	Ion exchange, H-bonding	Cheng et al. (2018)
Clay	Heat treatment at 573 K	—	44.29	—	6	—	Zhang et al. (2016c)
Scoria	HCl immersion for 24 h	—	—	—	—	Ion exchange, complexation	Asadi et al. (2018)
Porous nanohydroxyapatite	Organic template coprecipitation	25 nm	41.3	-	6.8	Lattice substitution, precipitation	Wimalasiri et al. (2021)
Hierarchical hydroxyapatite	Ca and phosphate hydrothermal at 393 K	2 μm	83.17	11.52	7.73	Electrostatic attraction, ion exchange	Gao et al. (2019)
NaP-hydroxyapatite	Hydrothermal with zeolite gel at 373 K	2 μm	45	13.7	—	Ion exchange	Zendehdel et al. (2017)
Hydroxyapatite	Aqueous double decomposition	—	—	—	—	Ion exchange	Mourabet et al. (2015)

#### 2.1.2 Rare Earth Metal Compounds

Compared to aluminum, the rare earth metals (cerium, titanium, lanthanum, etc.) have further affinity for fluoride due to the ability to stabilize in the +3 or +4 valence state with a few numbers of outermost electrons; therefore, sufficient empty orbitals are available for fluoride ions (Zhang K. et al., 2016). The solubility of rare earth metals is relatively limited over a wide pH range (Dhillon et al., 2016), so rare earth oxides/hydroxides have been increasingly investigated as substitution for aluminum in recent years. Among these, CeO_2_ readily forms oxygen vacancies and, therefore, has particularly high oxygen storage/release capacity with high adsorption capacity (Kullgren et al., 2014; Wu and Gong 2016; Kang et al., 2017). Kang et al. (2017) compared the physicochemical characteristics and adsorption performance of different morphologies of CeO_2_ (nanorods, octahedrons, and nanocubes) prepared under different hydrothermal conditions. The different morphologies of CeO_2_ were found to expose distinct crystalline surfaces and proportions of oxygen defects, leading to significant differences in fluoride adsorption capacity, with CeO_2_ nanorods having the largest *Q*_max_ (71.5 mg/g). However, rare earth metal oxides are costly, prone to agglomeration, and high leaching concentrations can be toxic to water (Yu et al., 2015).

#### 2.1.3 Magnesium Oxide

MgO is less dissolved, nontoxic, abundant in reserves compared to other metals, and has an affinity for fluoride (Jin et al., 2016), giving it an opportunity to be used. It has been reported that MgO has a high isoelectric point and relies on electrostatic attraction to adsorb fluoride (Suzuki et al., 2013). Y. Zhang et al. tested the zeta potential of hollow MgO spheres of *pH*_*PZC*_ = 10, which is the highest value reported. In order to improve the morphology, Z. Jin et al. used a typical solvothermal method followed by calcination to form porous MgO nanoplates with an increased maximum adsorption capacity from 115.5 mg/g to 185.5 mg/g. They suggested that the mechanism of adsorption mainly consists of ligand exchange between fluoride and hydroxyl groups and carbonates on the surface of MgO (Figure 1B). Thus, the presence of carbonate in the solution can affect the fluoride adsorption capacity of MgO.

The most significant problem concerning metal oxide/hydroxide nanoparticles is low structural stability and the tendency to leach in water causing secondary contamination (Lin et al., 2020).

### 2.2 Biopolymer Adsorbents

Biopolymers are the natural macromolecular materials derived from cellular or extracellular substances with properties such as biodegradability, nontoxicity, low waste generation, low leaching, biocompatibility, and hydrophilicity. The most researched fluoride removal biopolymer adsorbents in recent years include sodium alginate (SA), pectin, chitosan (CS), and carboxymethyl cellulose (CMC) (Araga and Sharma 2019). Hydrogels formed by chemical or physical cross-linking of biopolymers have hydrophobic, three-dimensional network structures, which are easier to separate compared to the powder state, making them an environment-friendly adsorbent.

#### 2.2.1 Sodium Alginate

**FIGURE 2:**
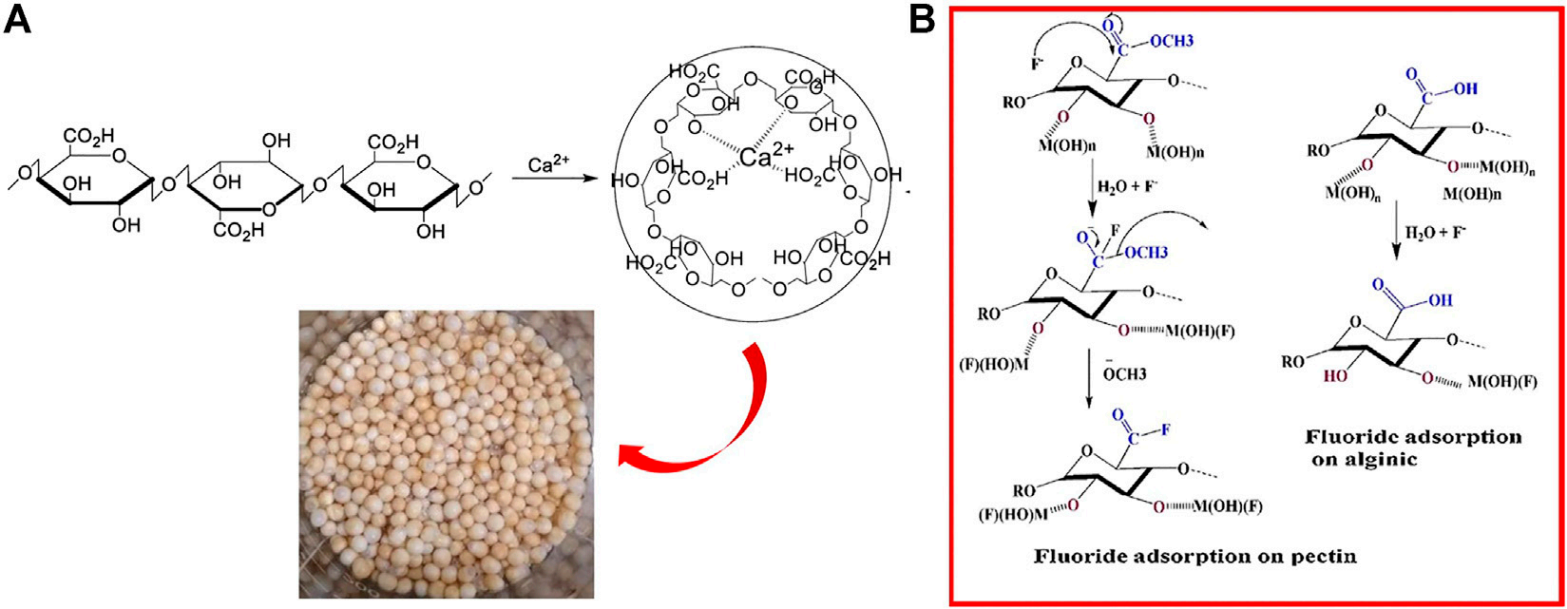
Ionic cross-linking procedure of sodium alginate **(A)** (Sharma et al., 2019) and fluoride adsorption mechanism of pectin **(B)** (Raghav and Kumar 2019).

Sodium alginate (SA) and pectin are both natural polysaccharides in colloidal form. Sodium alginate contains large numbers of -OH and -COOH groups on the main chain. The -COOH in the M unit is more bound by the surrounding electron cloud, while the -COOH in the G unit is arranged in the corner of the peak consisting of two adjacent carbon atoms; thus, G unit is more reactive (Wu T. et al., 2017). In the ionic cross-linking process (Figure 2A), when the dissolved colloidal sodium alginate is dropped into the solution of high-valent metal cations (Ca^2+^, Ce^3+^, Fe^3+^, Al^3+^, La^3+^, etc.), the high-valent cations in the solution will rapidly replace Na^+^ (Wu et al., 2016a). The embedded high-valent cations form ligand chelate crosslinks with the oxygen atoms in the carboxyl and hydroxyl groups of the G-units, which form irreversible hydrogel-like microbeads (Qiusheng et al., 2015). The thermal stability and acid resistance of sodium alginate are further improved after the formation of the gel, while some of the carboxyl functional groups are occupied by high-valent metal cations, so the active sites with an affinity for fluoride ions are increased. Huo et al. (2011) used ionic cross-linking to prepare lanthanum alginate with stable skeletal junctions. SEM showed cracks in the dense surface structure after adsorption, with *Q*_max_ = 197.2 mg/g.

#### 2.2.2 Pectin

Pectin is also rich in -COOH and -COOCH_3_ groups. The active sites of sodium alginate and pectin are essentially identical, the only difference being the presence of -COOCH_3_ in pectin (Sharma et al., 2019), whereas sodium alginate contains only -COOH. The ester group chelates better with metals through its lone pair of electron contribution. The carboxyl group is present in a dimeric form due to the conjugation effect, with the lone pair participating in the conjugation. Therefore, the ester group has a nucleophilic reaction to F^−^ (Figure 2B), providing more active sites, and the pectin should have a higher fluoride removal capacity in comparison (Raghav and Kumar 2019). SA and pectin hydrogels accomplish adsorption by exchanging hydroxyl groups in the structure with fluoride.

### 2.3 Carbon-Based Adsorbents

Carbon-based adsorbents have developed pore structures, large specific surface areas, stable chemical properties, easily adjustable surface properties, good regenerability, and widely available and general waste, which is of low cost with promising applications.

#### 2.3.1 Biochar

**FIGURE 3:**
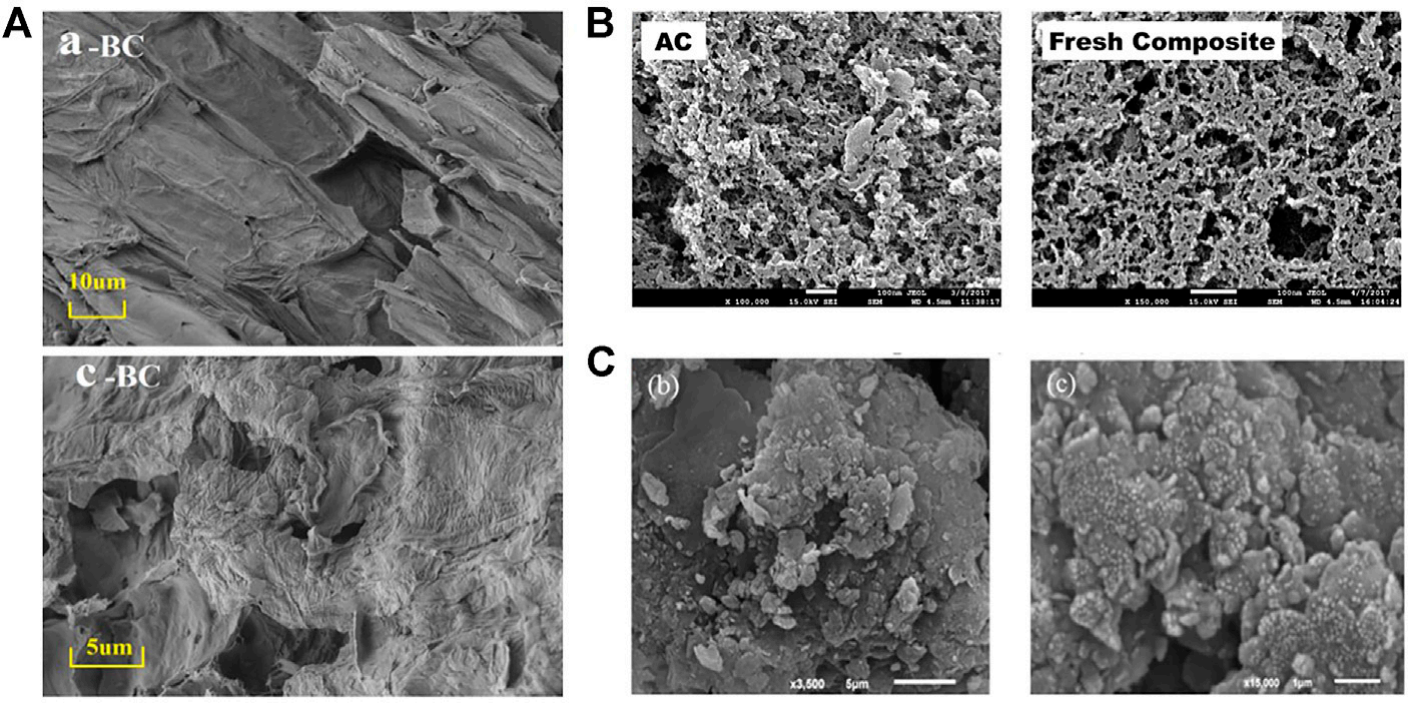
Rough surface of biochar **(A)** (Zhang X. et al., 2021), activated carbon **(B)** (Mullick and Neogi 2018), and clay **(C)** (Mobarak et al., 2018).

Biochar (BC) is made from waste biomass from a wide range of sources such as reed (Singh and Majumder 2018), rice husks (Yadav and Jagadevan 2020), straw (Angelin et al., 2021), teak peel, and algae. BC is a carbon-rich, fine-grained, porous, and highly aromatized material and well suited as an adsorbent for the resource utilization of waste. BC contains lignocellulosic components capable of effectively adsorbing fluoride (Yadav and Jagadevan 2020). The pyrolysis temperature is a key factor in controlling the number of functional groups on the surface of BC (Wang et al., 2021). Generally, biochar prepared by hydrothermal pyrolysis below 573 K is rich in oxygen-containing functional groups (-COOH, -OH, etc.) and has stronger ion exchange capacity (Naga Babu et al., 2020). As the pyrolysis temperature increases, the abundance of hydroxyl, amino, and carboxyl groups decrease and the degree of carbonation increases (Kumar et al., 2020). Biochar prepared at 673–973 K has developed porosity (Figure 3A) and thermal stability (Goswami and Kumar 2018). Brunson and Sabatini (2015) recorded a significant increase in specific surface area (from 0.9 m^2^/g to 327 m^2^/g) and surface zero charge point (from *pH*_*PZC*_ = 5.8 to *pH*_*PZC*_ = 9.4) when the pyrolysis temperature of charcoal was increased from 573 to 873 K. BC also contains minerals such as potassium, calcium, magnesium, and phosphorus, which can be complex with fluoride ions or precipitate. Lee et al. (2021) found that the shell biochar could contain up to 56.9% CaCO_3_, and when the pyrolysis temperature was raised to 1073 K, CaCO_3_ was converted to Ca(OH)_2_; the structure was more conducive to the adsorption of fluoride. The adsorption mechanism was outer-sphere complexation between Ca and F. Although shell biochar has low carbon content and small specific surface area (*S*_*BET*_ = 4.363 m^2^/g), the maximum fluoride adsorption capacity of their prepared shell biochar MCS-800 could reach 82.93 mg/g. The biochar obtained by pyrolysis alone has an average low adsorption effect but has the advantage of being easily modified (Wang et al., 2021).

#### 2.3.2 Activated Carbon

Activated carbon (AC) is usually made from coconut shells, hard cores, bamboo, coal, wood, and other raw materials (Collivignarelli et al., 2020), and the pyrolysis temperature is generally higher than 1173 K (Araga et al., 2019). After pyrolysis, further physical or chemical activation is required (Chen et al., 2019). Chemical activation has high activation yield but is highly corrosive to the equipment (Tomar et al., 2014). As shown in Figure 3B, AC has the advantage of high porosity and large specific surface area. Araga et al. (2017) and Bhomick et al. (2019) used KOH to activate mustard seed activated carbon and commercially available activated carbon, respectively, and the modified specific surface area reached 747 m^2^/g and 1,005 m^2^/g, respectively. The -OH group on the AC surface is protonated with fluoride at pH < *pH*_*PZC*_ (acidic media). Numerous studies have demonstrated that fluoride adsorption on AC consists of the mechanism for the deprotonation of -OH functional groups on carbon surfaces (He et al., 2020).

#### 2.3.3 Bone Char

Bone char is the charring product of animal bones and generally contains about 20% carbon and 80% hydroxyapatite (HAp) (Medellin-Castillo et al., 2014), with the content of each component varying slightly depending on the charring temperature. When the charring temperature is below 573 K, more organic matter remains in the bones, but the specific surface area and pore structure is not well developed (Zhou J. et al., 2019). The charring temperatures above 873 K may change the structure of the hydroxyapatite and also lead to reduction in fluoride adsorption capacity. The fluoride adsorption by bone char is reported to be mainly carried out by hydroxyapatite. Medellin-Castillo et al. (2014) found that fluoride in aqueous solutions was mainly adsorbed to HAp in bone char but not to other components. HAp in bone char contains most of the OH^−^ that can be replaced by F^−^; the adsorption mechanism includes ion exchange and chemical precipitation. The detailed mechanism of fluoride adsorption by HAp is described in the following section.

### 2.4 Other Types of Adsorbents

Other materials such as natural mineral clays (clay (Zhang S. et al., 2016), bentonite (Mudzielwana et al., 2017), etc), industrial solid waste (zeolite (Ghosal and Gupta 2018), etc), and hydroxyapatite are also used for fluoride adsorption. Natural clay (Figure 3C) contains the main compounds SiO_2_ and Al_2_O_3_ and has the chemical potential to adsorb fluoride (Guiza et al., 2019). Zeolite is an aqueous skeletal structure composed of aluminosilicate minerals with the lattice of many pores and channels that have the structural potential to adsorb fluoride. However, these two types of materials usually show weak fluoride adsorption capacities (Table 2) and are generally modified by chemical activation or metal loading.

**TABLE 2:** Adsorption conditions and performance of fluoride by four conventional adsorbents.

Adsorbents	Adsorption condition					Isotherm model	Regeneration performance	*Q*_max_ (mg/g)	Ref
	Initial *C*_*F*_^*−*^ (mg/L)	Adsorbent dose (g/L)	Reaction pH	Temperature (K)	Equilibrium time (min)				
Cactus-like amorphous alumina oxide microspheres	50	1	5–8	298	300	Langmuir	80% at 5th cycle	129.40	Kang et al. (2018)
Nano γ-alumina	8	1	4	303	120	Freundlich	80% at 5th cycle	32.00	Chinnakoti et al. (2016a)
Mesoporous micro alumina	80	0.5	5.5	298	60	Langmuir	—	26.00	Lanas et al. (2016)
Al_2_O_3_ nanoparticles	10	0.5	4–6	298	60	Langmuir	—	13.70	Hafshejani et al. (2017)
Amorphous porous-layered Al_2_O_3_	120	5	2–5	313	150	Langmuir	—	12.05	Yang et al. (2020)
Activated alumina	10	2	6–8	318	120	Freundlich	—	4.31	Dhawane et al. (2018)
Aluminum oxide/hydroxide	10	8	4–7	298	300	Langmuir	—	2.00	Rathore and Mondal (2017)
Cubical ceria nano-adsorbent	20	1	7	298	120	Langmuir	—	80.64	Dhillon et al. (2016)
CeO_2_ nanorods	50	0.5	3.5	298	1,500	Langmuir	—	71.50	Kang et al. (2017)
CeO_2_ octahedron	10	1	3–10	293	400	Langmuir	—	40.13	Zhang et al. (2016b)
CeO_2_ nanocubes	50	0.5	3.5	298	1,500	Langmuir	—	28.30	Kang et al. (2017)
CeCO_3_OH nanosphere	50	0.5	3.5	298	1,500	Langmuir	—	7.00	Kang et al. (2017)
Porous MgO nanoplates	20	1	2–11	298	180	Freundlich	—	185.50	Jin et al. (2016)
Hollow MgO spheres	10	1	3–11	298	250	Freundlich	—	182.40	Zhang et al. (2021b)
Microsphere-like MgO	100	1	3–9	298	300	Langmuir	40% at second cycle	166.70	Lee et al. (2017)
Pillar-like MgO	100	1	3–9	298	300	Langmuir	40% at second cycle	151.50	Lee et al. (2017)
γ-Fe_2_O_3_ nanoparticles	100	10	4.5	298	15	-	—	3.65	Jayarathna et al. (2015)
Trititanate nanotubes	10	0.5	2	298	10	Langmuir	—	58.60	Chinnakoti et al. (2016b)
TiO_2_	5	0.5	7	298	30	Langmuir	—	5.00	Zhou et al. (2019b)
Lanthanum alginate bead	10	1	4	298	1,440	Langmuir	—	197.20	Huo et al. (2011)
Biopolymer pectin and alginate	60	0.1	7	298	35	Langmuir	—	50.00	Raghav et al. (2019)
Porous zirconium alginate	20	1	2	303	1,200	Langmuir	—	27.95	Qiusheng et al. (2015)
Shell biochar	300	3.33	7	298	1,440	Langmuir	60% at third cycle	82.93	Lee et al. (2021)
Nanoscale rice husk biochar	5	1	7	303	60	Freundlich	—	21.70	Goswami and Kumar (2018)
Mustard ash biochar	5	2	2	298	150	Langmuir	30% at third cycle	4.42	Jadhav and Jadhav (2021)
Peanut shell biochar	10	8	7	298	120	Langmuir	—	3.66	Kumar et al. (2020)
Rhodophyta biochar	15	0.6	6	303	90	Freundlich	80% at fifth cycle	2.10	Naga Babu et al. (2020)
Rice husk biochar	4	5	6	303	360	Langmuir	-	1.86	Yadav and Jagadevan (2020)
Activated sugarcane ash	5	2	2	303	100	Langmuir	-	10.99	Mondal et al. (2016)
KOH-treated jamun seed	10	0.4	2.5	298	120	D-R	-	3.65	Araga et al. (2017)
KOH-treated activated carbon	5	3	4	303	100	Langmuir	-	2.52	Bhomick et al. (2019)
Activated carbon	380	2	3	298	—	—	50% at fifth cycle	1.15	Chen et al. (2019)
Coconut-shell carbon	4.4	10	2	323	180	Langmuir	—	0.36	Araga et al. (2019)
Chicken bone biochar	10	—	—	298	1,440	Langmuir	—	11.20	Herath et al. (2018)
Bone char	10	1	7	298	1,440	Langmuir	—	5.40	Medellin-Castillo et al. (2014)
Bovine bone biochar	20	5	8	298	—	Langmuir	50% at fourth cycle	5.05	Zhou et al. (2019a)
Kaolinite	100	1	7	298	150	Langmuir	—	125.00	Wang et al. (2017b)
Activated clay	30	1	5	298	80	Langmuir	—	75.76	Guiza et al. (2019)
Fly ash–paper mill lime mud	15	1.5	5	298	120	Langmuir	—	7.37	Ye et al. (2019)
Natural clay	5	1	6	301	120	Langmuir	—	3.74	Nabbou et al. (2019)
Natural pumice	3	0.7	3	298	50	Freundlich	—	1.17	Dehghani et al. (2016)
Natural zeolite	80	1	6–7	293	300	Freundlich	—	1.83	Cheng et al. (2018)
Clay	5	20	6	398	600	Langmuir	80% at sixth cycle	1.30	Zhang et al. (2016c)
Scoria	7	4	7	298	60	Freundlich	—	0.32	Asadi et al. (2018)
Porous nanohydroxyapatite	5	2	6.5	303	30	Langmuir	—	54.40	Wimalasiri et al. (2021)
Hierarchical hydroxyapatite	20	0.4	4	298	10	Langmuir	—	29.82	Gao et al. (2019)
NaP-hydroxyapatite	5	3	4.5	298	50	Langmuir	—	11.95	Zendehdel et al. (2017)
Hydroxyapatite	15	0.7	7.5	303	60	Langmuir	—	3.12	Mourabet et al. (2015)

#### 2.4.1 Hydroxyapatite

Hydroxyapatite [(Ca_10_(PO_4_)_6_(OH)_2_, HAp] is also a promising inorganic material for fluoride adsorption, with excellent biocompatibility, stability, and mechanical properties. Due to its unique crystal structure, HAp has a porous surface, large specific surface area, and high ion exchange capacity. The hydroxyl group in HAp is prone to rapid exchange with anion and has a strong binding capacity with fluoride (Raghav et al., 2018). F^−^ replaces OH^−^, fills in the lattice of HAp forming insoluble fluorapatite (FAp), and OH^−^ is released into solution. When high concentrations of fluoride ions are present in the solution, Ca^2+^ in HAp reacts with F^−^ forming CaF_2_ precipitate, and phosphate is correspondingly released into the solution. Various forms of HAp have been reported for fluoride adsorption in water, such as nano-hydroxyapatite (Mourabet et al., 2015; Zendehdel et al., 2017), porous hydroxyapatite (Nijhawan et al., 2020), and layered hollow hydroxyapatite (Gao et al., 2019). The mechanism of fluoride adsorption by HAp mainly consists of the following: 1) electrostatic attraction by the surface of HAp to F^−^. 2) Anion exchange between OH^−^ or PO_4_^2-^ and F^−^. 3) Complexation reaction of Ca^2+^ with F^−^ ligates and forms surface precipitation. 4) F^−^ can also form hydrogen bonds with OH^−^ in the HAp lattice.

## 3 New Composite Adsorbents Obtained From Metal Modification

Over long periods of use and development, traditional adsorbents have gradually revealed the unique application value and drawbacks. Compared to the exploitation of novel adsorbents, research tends more to synthesize complexes of two or more adsorbents to produce synergistic fluoride adsorption. The synthesis of metal modifications to other types of adsorbents accounts for the majority.

### 3.1 Multi-Metal Oxide/Hydroxide Adsorbents

Different metal oxide adsorbents have their individual strengths and weaknesses for fluoride removal, so recently there have been research studies using multi-metal oxide/hydroxide adsorbents (Chen et al., 2018). Compared to conventional metal oxides, various valence cations are often present in one multi-metal oxide, providing more chemisorption sites (Raghav and Kumar 2018). The tunability of the chemistry of each element ensures an abundance of active sites, and the components can be adjusted to each other, possessing different outstanding properties and therefore having unique quantum coupling effect and synergistic effects, resulting in more than doubling or tripling of the adsorption capacity. There are two common types of multi-metal oxide adsorbents. One is prepared by compounding each metal element in a certain ratio (Wu K. et al., 2017) such as layered double/triple hydroxides (Wu P. et al., 2017), and the other is to modify one metal oxide with others; the ones mostly reported are modified alumina (He et al., 2019) or magnetic iron oxides.

#### 3.1.1 Layered Double/Triple Hydroxides

A series of layered double/triple hydroxides (LDHs) have a high affinity for anions with high ion exchange capacity and high adsorption volume, which are often used as anion exchangers and trapping agents. LDHs are two-dimensional layered materials whose structural formula can be expressed as ${\left[M_{1-x}^{2+}\;M_{x}^{3+}{\left(OH\right)}_{2}\right]}^{x+}{\left(A^{n-}\right)}_{x/n}\cdot mH_{2}O$ , where M^2+^ is the positive divalent metal ion (Mg^2+^, Cu^2+^, Ni^2+^, Zn^2+^, etc.), M^3+^ is the positive trivalent metal ion (Fe^3+^, Al^3+^, La^3+^, Ce^3+^, etc.), and A is the interlayer anion (Cl^−^, CO_3_^2-^, NO_3_^−^, etc.). LDH consists of positively charged main lamellae and negatively charged interlayer ions. The lamellar structure of LDH is longitudinally stable (Wu et al., 2015). This lamellar structure facilitates adequate contact between the metal sites and the fluoride ions during adsorption and accelerates the charge transfer at the interface. The surface of the main layer is rich in hydroxyl functional groups, which bind to cations in different ways such as electrostatic gravitational forces and hydrogen bonding, providing a large anion exchange capacity with fluoride ions. The synthesis of triple hydroxide by doping of layered double hydroxide with high-valent metal cations has been shown to be effective in enhancing its adsorption activity. When the highly valent cation M^3+^ replaces M^2+^, the main lamellae are positively charged and therefore require the interlayer anions to be negatively charged to balance the overall charge (Hongtao et al., 2018). The addition of rare earth metals has been reported to further enhance the affinity with fluoride. Wu P. et al. (2017) introduced La into Mg/Fe LDH to form Mg/Fe/La hydrotalcite-like compounds with layered porous structure, which significantly enhanced the fluoride adsorption capacity of Mg/Fe LDH.

#### 3.1.2 Metal-Modified Magnetic Iron Oxides

**FIGURE 4:**
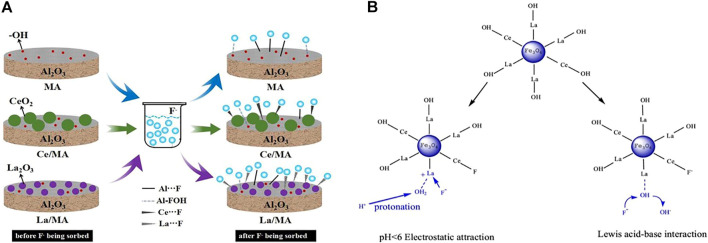
Schematic principle of modification on Al_2_O_3_ **(A)** (He et al., 2019) and Fe_3_O_4_ **(B)** (Han et al., 2019) by La, Ce, and fluoride adsorption mechanism.

Al- and Fe-based oxides are mostly doped or load-modified by another metal. Metal-modified Al_2_O_3_ generally adsorbs fluoride by complexation and ion exchange (Figure 4A). Iron oxides (Fe_3_O_4_ or γ-Fe_2_O_3_), in addition to adsorption advantages, provide strong magnetic properties and large magnetic response with easy separability and controllability; thus, there have been more reported recently. Magnetic iron oxide nanoparticles can be used directly for fluoride adsorption or as a nucleus material for core-shell particles. The magnetic core particles are generally combined with metal oxide nano-shells by methods such as surface coating to ensure stronger magnetic response, more functional groups, and better properties (Zhang C. et al., 2016). Recent research has mostly used rare earth metals to modify magnetite. Rare earth ions are greater in radius than other elements in the iron oxides; doping with the appropriate amount of rare earth elements to replace some of the other elements in the iron oxides with a smaller ion radius distorts the lattice and can improve the physical activity. Most importantly, the hard Lewis acid nature of the rare earth metal ions (especially La) has a strong affinity for fluoride (Figure 4B) (Han et al., 2019). Ce can promote the dispersion of nanoparticles, giving the adsorbent a larger specific surface area, pore volume, and more active functional sites. Correspondingly, magnetic particles can attenuate the agglomeration effect of rare earth metal oxides, reducing the amount of precious metals used and improving the separation characteristics by magnetic assistance to reduce residues in water and avoid rare earth metal toxicity (Abo Markeb et al., 2017). Han et al. (2019) used VSM tests to show that Fe_3_O_4_ and Fe_3_O_4_@La-Ce were both superparamagnetic, and it was easy to separate the particles from the solution using the external magnetic field. The adsorption amount of Fe_3_O_4_@La-Ce was increased up to 20 times compared to Fe_3_O_4_.

### 3.2 Metal-Biopolymer Adsorbents

Large-scale applications for fluoride removal in aqueous systems require the development of composite hydrogel materials with good mechanical properties and stability. For hydrogels with poor adsorption properties, the cross-linking of composites by cementing other high performance adsorbent materials onto biopolymers can effectively reduce the degradation of properties. The preparation of biopolymer-based composites is divided into three types: 1) doping of metals/metal oxides (Sapna et al., 2018), 2) blending with inorganic materials (Wang et al., 2020), and 3) mixing between polymeric organic substances (Preethi and Meenakshi 2018). This section summarizes the doping by metal oxide modification studies.

#### 3.2.1 Metal-Doped Alginate/Pectin

**FIGURE 5:**
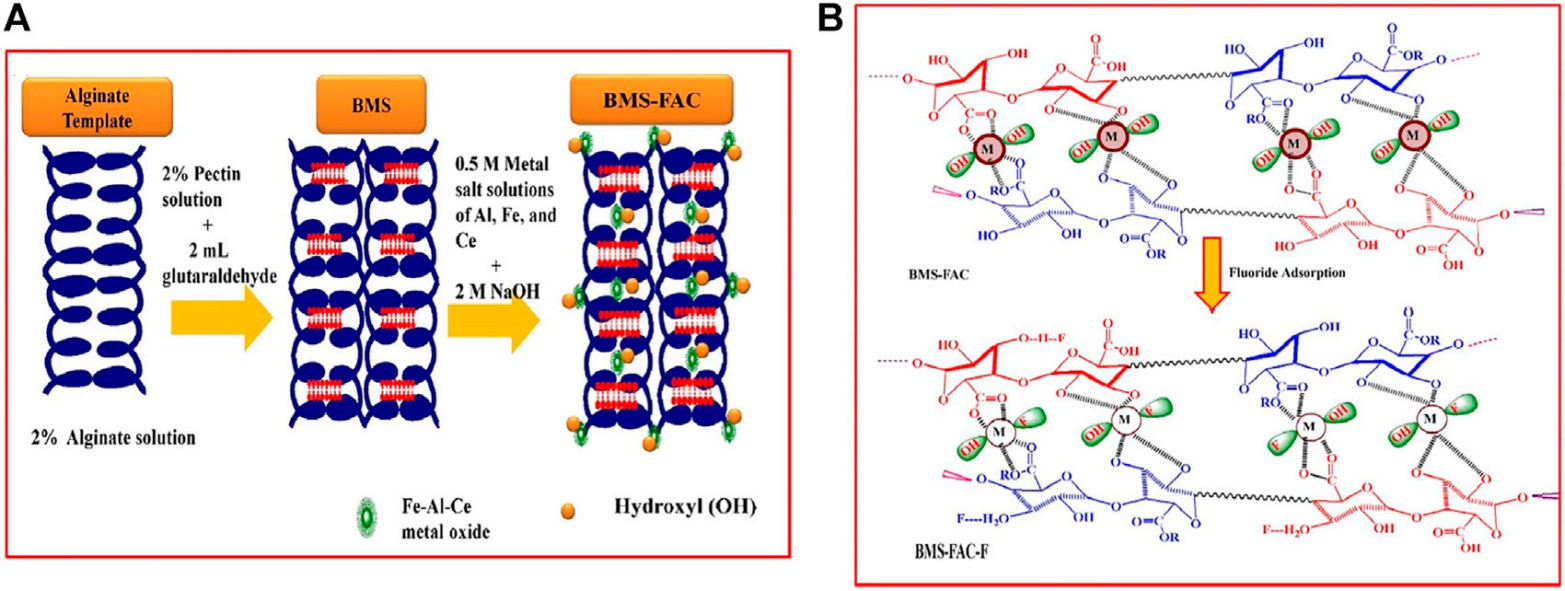
Schematic principle **(A)** and fluoride adsorption mechanism **(B)** of multi-metal–modified sodium alginate and pectin (Raghav et al., 2019).

The biocompatibility and biodegradability of natural polymeric materials make sodium alginate and pectin effective substrates for the incorporation of multivalent metal ions. Studies have reported to dope SA with metals; co-mingling and cross-linking to form a stable gel structure can improve both the stability and mechanical properties of SA (Wu T. et al., 2017), while having an anchoring effect on metal oxides, reducing the agglomeration and leaching of metal oxides and maximizing the adsorption properties (He et al., 2020). Furthermore, the doping of metals can increase the metal active sites in the porous structure and combine the properties of organic and inorganic components to improve the adsorption capacity (Zhao et al., 2021). Mono and multi-metal doping options are available. Recent research has focused on the doping of sodium alginate and pectin with multi-metals. Compared to monometals, the multi-metals provide an enhanced abundance of active sites as mentioned earlier. In addition, the multi-metals used in the studies tend to be the composite of +2 valent and higher valent cations. The addition of +3 and +4 valent metal ions, especially rare earth metals, can improve the stability, recyclability, and adsorption capacity. Raghav and Kumar (2019) obtained a high adsorption capacity (Table 3) for all the composite hydrogels prepared by SA and pectin embedding Fe-Al-Ni (285 mg/g and 200 mg/g) and SA/pectin co-embedding Fe-Al-Ce (142.9 mg/g) (Raghav et al., 2019). Figure 5 shows the reaction process of multi-metal–modified SA and pectin and the exchange sites for fluoride adsorption.

**TABLE 3:** Adsorption conditions and performance of fluoride by muti-metal and metal-biopolymer composite adsorbents.

Adsorbents	Adsorption condition					Isotherm model	Regeneration performance	*Q*_max_ (mg/g)	Ref
	Initial *C*_*F*_^*−*^ (mg/L)	Adsorbent dose (g/L)	Reaction pH	Temperature (K)	Equilibrium time (min)				
Mn–Al binary metals	380	—	7	298	720	Langmuir	-	94.83	Wu et al. (2017a)
Ce–Zn binary metals	10	0.15	3–7	298	45	Langmuir	68% at sixth cycle	64.66	Dhillon et al. (2017)
Ce–Ti oxide	10	1	7	298	—	Langmuir	—	44.37	Abo Markeb et al. (2017)
Mg/Fe-LDHs	30	4	7	298	150	Langmuir	—	28.65	Wu et al. (2015)
Fe-La	10	1	6	298	60	Langmuir	—	27.42	Wang et al. (2018c)
La/MA	10	2	6	298	360	Sips	70% at fifth cycle	26.45	He et al. (2019)
Fe–Ag magnetic oxide	10	0.5	3	298	20	Langmuir	85% at sixth cycle	20.57	Azari et al. (2015)
La-modifying Fe_3_O_4_	5	10	7.4	303	600	Langmuir	—	1.51	García-Sánchez et al. (2016)
Al-modifying Fe_3_O_4_	5	10	6.6	303	600	Langmuir	—	1.42	García-Sánchez et al. (2016)
Ca-Mg-Zr oxide	100	0.5	7	298	160	Freundlich	70% at fifth cycle	370.37	Wang et al. (2022)
Ce-Ti@Fe_3_O_4_	10	1	7	298	15	Langmuir	93% at fifth cycle	91.04	Abo Markeb et al. (2017)
Fe_3_O_4_@La-Ce	10	0.5	4	303	60	Freundlich	—	56.80	Han et al. (2019)
Fe_3_O_4_@Fe-Ti	4	1	7	298	2	Langmuir	77% at ninth cycle	41.80	Zhang et al. (2016a)
Fe-Mg-La	10	0.1	7	298	300	Langmuir	90% at third cycle	185.90	Yu et al. (2015)
Al-Zr-La	200	0.5	3	308	500	Langmuir	—	90.48	Zhou et al. (2018)
Mg/Fe/La	5	0.5	7	308	100	Langmuir	57% at fifth cycle	59.34	Wu et al. (2017b)
Fe-Mg-La	20	1	7	298	360	Langmuir	—	40.40	Chen et al. (2018)
Mg-Al-Fe LDH	2	1.5	6	298	600	Sips	—	20.00	Hongtao et al. (2018)
Fe-Al-Ce-Ni	10	0.4	5	303	50	Freundlich	50% at sixth cycle	250.00	Raghav and Kumar (2018)
SA-Ca@Fe/La/Ni	10	30	5	303	30	Freundlich	55% at fifth cycle	333.00	Sapna et al. (2018)
Pectin-Fe/Al/Ni	10	0.4	7	318	90	Freundlich	86% at fifth cycle	285.00	Raghav and Kumar (2019)
Alginate-Fe/Al/Ni	10	0.4	7	298	90	Langmuir	84% at fifth cycle	200.00	Raghav and Kumar (2019)
SA/pectin-Fe/Al/Ce	60	0.1	7	298	35	Halsey	65% at ninth cycle	142.90	Raghav et al. (2019)
SA/CMC-Ca-Al	40	-	2	298	600	Langmuir	—	101.40	Wu et al. (2016b)
SA-Mg/Fe oxide	10	10	7	298	600	Langmuir	80% at third cycle	32.31	Wu et al. (2017c)
SA-Mg/Al/Zr	40	2.5	6	303	1800	Freundlich	—	31.72	Wang et al. (2017a)
SA-Mg/Al/Ce	40	5	6	303	3,600	Freundlich	65% at third cycle	26.12	Wang et al. (2018a)
Pectin Fe bead	10	2	5	298	600	Freundlich	—	20.00	Sharma et al. (2019)
CS-Ce	30	0.3	3	293	400	Langmuir	80% at fourth cycle	153.00	Zhu et al. (2017)
Fe_3_O_4_/CS/Al(OH)_3_	10	0.1	5	298	60	Langmuir	—	76.63	Hu et al. (2018)
Fe-Al-Mn@CS	6	0.5	7	298	160	Langmuir	—	40.50	Chaudhary et al. (2021)
Rare earth CS bead	10	2	5	298	480	Freundlich	70% at seventh cycle	22.35	Liang et al. (2018)
La^3+^ magnetic CS	10	2	5	298	480	Langmuir	40% at seventh cycle	20.53	—
Zr-CS bead	20	1	7	303	80	Freundlich	—	17.47	Prabhu and Meenakshi (2015)
La-CS bead	20	1	7	303	80	Freundlich	—	14.49	—
Ce-CS bead	20	1	7	303	60	—	—	11.50	—
Al-CS bead	20	1	7	303	40	—	—	7.45	—
Fe_3_O_4_@TiO_2_-CS	2	0.4	5	298	30	Langmuir	75% at sixth cycle	14.62	Sadeghi et al. (2019)
Fe_3_O_4_-CS	5	1	7	293	60	Freundlich	88% at fifth cycle	9.26	Mohseni-Bandpi et al. (2015)
La-CS/β cyclodextrin	10	2	7	303	30	Freundlich	56% at fifth cycle	8.14	Preethi and Meenakshi (2018)
Ce-cellulose nanobead	2.5	1	3	303	50	Langmuir	82% at fifth cycle	39.88	Sarkar and Santra (2015)
CMKGM-La-Al	40	2	2	40	120	Langmuir	-	20.37	Wu et al. (2016a)

#### 3.2.2 Metal-Doped Chitosan

Chitosan (CS) is an N-deacetylated derivative of the natural polysaccharide chitin and is rich in free amino acids. The -NH_2_ group in chitosan is more reactive (Zhu et al., 2017), easy to be chemically modified (Chaudhary et al., 2021), and exhibits high adsorption potential. Despite the numerous advantages such as biodegradability, biocompatibility, flexibility, hydrophilicity, and versatility, CS tends to be readily soluble in acidic solutions and has a weak chemical resistance (Dong and Wang 2016), especially in column continuous flow adsorption. Current research into the adsorption of fluoride ions by CS is also mostly metal-doped, but unlike sodium alginate and pectin, the modification of CS is more oriented toward monometallic impregnation followed by cross-linking using glutaraldehyde (Table 4). The size of the beads formed is much smaller, typically in the micron range (Prabhu and Meenakshi 2015). One of the top research hotspots is the magnetic modification of Fe_3_O_4_, mainly because the hydroxyl group on the surface of Fe_3_O_4_ can interact with the amino and hydroxyl groups of CS through hydrogen bonding (Sadeghi et al., 2019), enabling CS to remain stable under acidic conditions. The CS composite adsorbent is also endowed with magnetic ease of separation properties (Mohseni-Bandpi et al., 2015). Hu et al. (2018) obtained nano-microsphere Fe_3_O_4_/CS/Al(OH)_3_ beads by facile impregnation, which can rapidly accomplish high capacity adsorption of fluoride and rapid sedimentation under low magnetic fields.

**TABLE 4:** Summary of modification methods, characteristics, and adsorption mechanisms of muti-metal and metal-biopolymer composite adsorbents.

Adsorbents	Modification method	Dimension	SBET (m2/G)	Aperture (nm)	pHPZC	Adsorption mechanism	Ref
Mn–Al binary metals	Oxidation and coprecipitation	—	43	0.33	8.7	Surface complexation	Wu et al. (2017a)
Ce–Zn binary metals	Coprecipitation, calcination at 873 K	22.4 nm	499	15	6	Ion exchange	Dhillon et al. (2017)
Ce–Ti oxide	Coprecipitation	1–2 nm	—	—	—	Ion exchange	Abo Markeb et al. (2017)
Mg/Fe-LDHs	Coprecipitation, hydrothermal at 543 K	100 nm	—	—	10.42	Ion exchange	Wu et al. (2015)
Fe-La	Coprecipitation, hydrothermal at 423 K	—	113	21.78	8.5	Ion exchange	Wang et al. (2018c)
La/MA	Impregnation, calcination at 673 K	—	237	4.81	10.2	Electrostatic attraction, chemisorption	He et al. (2019)
Fe–Ag magnetic oxide	Coprecipitation	5 nm	254	0.13	6.03	Ion exchange	Azari et al. (2015)
La-modifying Fe3O4	Lanthanum hydroxide soaking	—	6	—	>10	Electrostatic attraction	García-Sánchez et al. (2016)
Al-modifying Fe3O4	Aluminum hydroxide soaking	—	5	—	>10	Electrostatic attraction	García-Sánchez et al. (2016)
Ca-Mg-Zr oxide	Hydrothermal, calcination at 923 K	—	119	14.03	11.5	Electrostatic attraction, ion exchange	Wang et al. (2022)
Ce-Ti@Fe3O4	Coprecipitation	15 nm	—	—	—	Ion exchange	Abo Markeb et al. (2017)
Fe3O4@La-Ce	Coprecipitation	—	40	20.3	6	Ligand exchange, electrostatic attraction	Han et al. (2019)
Fe3O4@Fe-Ti	Precipitation of Fe3O4@Fe-Ti, granulation	10 μm	99	15.3	-	Ion exchange	Zhang et al. (2016a)
Fe-Mg-La	Coprecipitation	40 μm	—	—	6.3	Ion exchange	Yu et al. (2015)
Al-Zr-La	Coprecipitation	—	36	—	8.4	Electrostatic attraction, ion exchange	Zhou et al. (2018)
Mg/Fe/La	Hydrothermal, calcination at 873 K	—	59	22.3	—	Surface complexation, ion exchange	Wu et al. (2017b)
Fe-Mg-La	Coprecipitation	65 nm	78	30	8.8	Ligand exchange	Chen et al. (2018)
Mg-Al-Fe LDH	Coprecipitation	—	130	24.47	—	Interlayer ion exchange	Hongtao et al. (2018)
Fe-Al-Ce-Ni	Coprecipitation, calcination at 873 K	—	184	51.43	6.2	Ion exchange, electrostatic attraction	Raghav and Kumar (2018)
SA-Ca@Fe/La/Ni	Fe-La-Ni oxides mixing SA, CaCl2 cross-linking	1–2 mm	257	10.4	7	Ion exchange, H-bonding	Sapna et al. (2018)
Pectin-Fe/Al/Ni	Aerogel formation by coprecipitation, freezing	886 nm	275	0.15	—	Isomorphic substitution	Raghav and Kumar (2019)
Alginate-Fe/Al/Ni	Aerogel formation by coprecipitation, freezing	914 nm	96	0.13	—	Isomorphic substitution	Raghav and Kumar (2019)
SA/pectin-Fe/Al/Ce	Fe-Al-Ce coprecipitation with pectin and alginate	—	275	—	7.17	Ion exchange, H-bonding, complexation	Raghav et al. (2019)
SA/CMC-Ca-Al	SA/CMC mixing, Ca2+ cross-linking, Al3+ soaking	2–2 mm	—	—	—	Coordination reaction	Wu et al. (2016b)
SA-Mg/Fe oxide	Mg/Fe oxide mixing SA, CaCl2 cross-linking	1 mm	—	—	10.52	Ligand exchange, electrostatic attraction	Wu et al. (2017c)
SA-Mg/Al/Zr	Mg-Al-Zr oxide mixing SA, CaCl2 cross-linking	1 mm	—	—	—	Ion exchange, electrostatic attraction	Wang et al. (2017a)
SA-Mg/Al/Ce	Mg-Al-Ce oxide mixing SA, CaCl2 cross-linking	—	—	—	—	Ion exchange	Wang et al. (2018a)
Pectin Fe bead	Grafting, FeCl3 impregnation	43 nm	—	—	—	Ligand exchange	Sharma et al. (2019)
CS-Ce	Coprecipitation, glutaraldehyde cross-linking	200 nm	17	—	5.3	Electrostatic attraction, ligand exchange, and complexation	Zhu et al. (2017)
Fe3O4/CS/Al(OH)3	AlCl3 mixing, Fe3O4 NP adding	200 nm	—	—	—	Electrostatic attraction, complexation	Hu et al. (2018)
Fe-Al-Mn@CS	Coprecipitation	—	42	—	—	—	Chaudhary et al. (2021)
Rare earth CS bead	Rare earth mixing, Fe3O4 adding, cross-linking	—	21	7.92	5	Ligand exchange	Liang et al. (2018)
La3+ magnetic CS	La mixing, Fe3O4 adding, cross-linking	—	17	8.15	5	Ligand exchange	—
Hyper-branched CS beads	Glutaraldehyde cross-linking, Zr, La, Ce, Al solution immersion	1.7 mm	3	—	7	Electrostatic attraction, ligand	Prabhu and Meenakshi (2015)
Fe3O4@TiO2-CS	Fe3O4@TiO2 impregnation CS	—	—	—	6	Electrostatic attraction, H-bonding	Sadeghi et al. (2019)
Fe3O4-CS	FeCl3 impregnating CS, coprecipitation	0.15 mm	499	3.4	7	—	Mohseni-Bandpi et al. (2015)
La-CS/β cyclodextrin	Mixing, 5% glutaraldehyde cross-linking	—	—	—	4.56	Electrostatic attraction, H-bonding	Preethi and Meenakshi (2018)
Ce-cellulose nanobead	Impregnation	45 nm	—	—	—	Ion exchange	Sarkar and Santra (2015)
CMKGM-La-Al	La, Al mixed solution cross-linking	-	—	—	—	Ion exchange, electrostatic attraction	Wu et al. (2016a)

### 3.3 Metal-Carbon Adsorbents

Carbon-based adsorbents have the advantage of large specific surface area and rich pore structure (Shang et al., 2022), but they have low adsorption capacity for fluoride removal alone and require some modification. Research on carbon composites has focused on the doping or surface loading of carbon with nano-metal oxides/hydroxides (Dehghani et al., 2018). The affinity between fluoride ions and highly valent cations such as Al^3+^, Fe^3+^, Ca^2+^, and Mg^2+^ can improve the selectivity of carbon to fluoride. When the two are compounded, on the one hand, the metal nanoparticles provide a large number of active sites (Mohanta and Ahmaruzzaman 2018) to compensate for the absence of functional groups that can interact with fluoride ions after high temperature carbonization. On the other hand, the carbon-based adsorbent has large specific surface area and pores, which can act as carriers and dispersants to avoid agglomeration of the metal nanoparticles (Cai et al., 2022). More individual metal loadings are used, and multi-metal modifications are also available. More active sites enhance the adsorption performance, and the modified adsorbent surface is richer in specific types of adsorption sites, which may further increase the adsorption capacity.

#### 3.3.1 Metal-Modified Biochar

The ability of biochar to remove pollutants is greatly influenced by the nature of the raw material, preparation technology, and pyrolysis conditions. Unsuitable pyrolysis conditions tend to under-carbonize or over-carbonize BC, so the adsorption performance of unmodified BC is limited. The raw biochar has a relatively poor adsorption effect on anions as the negative charge occupies the majority of the functional groups (Mei et al., 2020). Highly valent metal cations can provide sufficient positive charge to effectively alter surface physicochemical properties (Wang et al., 2019c). AlCl_3_ has been reported to generally increase the anion exchange capacity in all BC. Brunson and Sabatini (2015) studied the changes in charcoal surface area and surface chemistry following aluminum nitrate impregnation and found that the aluminum modification reduced the zero charge point of the charcoal in water (from *pH*_*PZC*_ = 9.6 to *pH*_*PZC*_ = 5.7) but significantly increased the adsorption capacity. Rare earth metal ions such as Ce^3+^, Zr^4+^, and La^3+^ are more alkaline, have a relatively low ionic potential, and show strong tendency to dissociate hydroxyl groups into ions. The possibility of ionic exchange with F^−^ is higher and the affinity is stronger. Habibi et al. (2019) modified woody BC with LaCl_3_ and showed that the maximum adsorption capacity of 164.23 mg/g and adsorption equilibrium could be reached within 30 min. They concluded that H^+^ in functional groups such as carboxyl and sulfate groups on the surface of BC may exchange with La^3+^ ions. The presence of La^3+^ increased the adsorption mechanism with Lewis acid–base interaction and ion exchange. The F^−^ adsorption rate of the adsorbent was still 80% at fifth recycling, indicating that the rare earth metals loaded on the BC are not easily leached. The modification of iron oxides can confer magnetic properties to BC, improving the separation and recovery performance. BC has good electrical conductivity, which is conducive to electron transfer and reduction of Fe^3+^. The stronger synergistic effect can further promote the fluoride adsorption performance (Wang et al., 2019c).

#### 3.3.2 Metal-Modified Activated Carbon

Activated carbon has large specific surface area and pores, which can act as carriers and dispersants to avoid agglomeration of the metal nanoparticles (Cai et al., 2022). More individual metal loadings are used, and multi-metal modifications are also available. More active sites enhance the adsorption performance, and the modified adsorbent surface is richer in specific types of adsorption sites, which may further increase the adsorption capacity. Li et al. (2018) precipitated Ti(OH)_4_ on the surface of AC, which further increased the specific surface area of Ti-AC to 1700 m^2^/g, providing more adsorption sites for fluoride ions. They confirmed that the adsorption capacity of Ti-AC was produced by Ti(OH)_4_ loaded on AC. The saturation adsorption capacity of Ti(OH)_4_ in Ti-AC was 62.1 mg/g, which was much higher than that of Ti(OH)_4_. It has also been reported that the loading of different metal oxides/hydroxides can form new functional groups on the AC surface with high affinity for fluoride adsorption, significantly improving the adsorption efficiency. A et al. used ultrasonically assisted polymetallic impregnation of AC (Mohanta and Ahmaruzzaman 2018; Mullick and Neogi 2018). The specific surface area decreased after modification, but the *pH*_*PZC*_ increased to 11.9 and the adsorption capacity increased by four times compared to monometallic impregnation (Mullick and Neogi 2019).

#### 3.3.3 Metal—Other Types of Adsorbents

Graphene oxide (GO) is a two-dimensional honeycomb carbon nanomaterial formed by the close packing of carbon atoms in a sp-hybridization pattern (Kanrar et al., 2016). GO carries various functional oxygen-containing groups (such as -OH, -COOH, C=O, and -CH(O)CH-) and provides active sites to connect to other substances (Jeyaseelan et al., 2021). GO generally adsorbs fluoride through electrostatic attraction, π-π stacking, and hydrogen bonding. It has been reported to have a huge theoretical specific surface area (up to 2,630 m^2^/g) (Mohan et al., 2017) and can be an excellent host for metal nanoparticles. In turn, nanometallic particles provide structural rigidity by inhibiting the restacking of different layers of GO and provide a higher surface area and many active centers (Mohan et al., 2017). Mohan et al. (2017) hydrothermally synthesized ZrO_2_/GO with *S*_*BET*_ = 632 m^2^/g. The fixed-bed continuous flow experiments showed that the desorption elution efficiency of the adsorption column regenerated with 10% NaOH solution was greater than 95% for F^−^ within three cycles, indicating the role of the ion exchange mechanism in the adsorption of F^−^. Nehra et al. (2019) hydrothermally synthesized TiO_2_/GO with a maximum fluoride adsorption capacity of 342 mg/g, which is the highest reported capacity available. Ti^4+^ forms strong bonds with the oxygen-containing functional groups of GO by electrostatic attraction and reacts with NaOH on the GO side to form basic titanium hydroxide on the GO layer. The adsorption mechanism for fluoride consists of a complexation reaction with Ti and F and an ion exchange between OH^−^ and F^−^.

### 3.4 Multiple Types of Metal-Modified Composite Adsorbents

Studies have also reported on composite adsorbents synthesized from three or more types of materials, with combinations of metal-modified biopolymers and inorganic materials making up the bulk of the adsorbents (Table 5). The compound of metallic, inorganic, and biomaterials effectively combine the advantages of different types and can yield further synergistic effects. He et al. (2018) achieved a maximum adsorption capacity of 288.96 mg/g for yttrium-based GO/SA hydrogels prepared by sol–gel. Wei et al. (2022) mixed reed biomass powder with SA, cross-linked with CeCl_3_ solution, and then calcined to obtain cerium alginate biochar beads. The composite adsorbent RBM-Ce has greatly improved the maximum adsorption capacity, *S*_*BET*_, *pH*_*PZC*_, and stability compared to individual components.

**TABLE 5:** Summary of modification methods, characteristics, and adsorption mechanisms of metal-modified carbon and other adsorbents.

Adsorbents	Modification method	Dimension	*S*_*BET*_ (m^2^/G)	Aperture (nm)	*pH*_*PZC*_	Adsorption mechanism	Ref
Wood biochar-La	Impregnation, pyrolysis	0.8 mm	165	3.91	6.6	Ion exchange	Habibi et al. (2019)
Al-modified corn biochar	Pyrolysis at 623 K, coprecipitation	—	1	410	2	Ion exchange	Zhang et al. (2021a)
MgO shell biochar	Impregnation, one-step calcination	0.5 μm	182	2–10	—	Electrostatic attraction, complexation	Wan et al. (2019)
Pomelo peel BC-La	Impregnation, calcination at 1073 K	—	269	—	5.8	Ion exchange	Wang et al. (2018b)
ZrO_2_-seed shell biochar	One-step impregnation and calcinationT	—	—	—	4.45	Ion exchange	Mei et al. (2020)
Magnetic biochar	Charring, impregnation-pyrolysis	100 μm	494	0.3	11	Electrostatic attraction, H-bonding	Bombuwala Dewage et al. (2018)
Mg-Mn-Zr AC	Ultrasound impregnation, coprecipitation	—	834	2.43	11.9	Electrostatic attraction, ion exchange	Mullick and Neogi (2019)
Zr-impregnated AC	Ultrasonic impregnation	14 μm	1,104	2.30	5.03	Electrostatic attraction	Mullick and Neogi (2018)
La-functionalized AC	Impregnation, rotary evaporation, heat	0.5 mm	367	0.68	7.3	Ligand exchange, electrostatic attraction	Merodio-Morales et al. (2019)
Activated carbon@SnO_2_	Ultrasound impregnation, precipitation	—	126	3.54	3	Ion exchange, physical adsorption	Mohanta and Ahmaruzzaman (2018)
Ce-containing bone char	Impregnation, heat treatment	0.7 mm	—	—	—	Electrostatic attraction, ion exchange	Zúñiga-Muro et al. (2017)
Magnetic bone biochar	Impregnated biomass, calcination	—	42	17.45	2.4	Ion exchange	Zhou et al. (2019a)
Graphene oxide with Ti	Hydrothermal at 453 K, calcination	—	278	2.55	7	Electrostatic attraction, ion exchange	Nehra et al. (2019)
Al-polyacrylic acid	Impregnation	—	44	84.63	6	Electrostatic attraction, ion exchange	Xu et al. (2017)
CeO_2_@SiO_2_ microsphere	Coprecipitation	117 μm	86	25–97	3.9	Electrostatic attraction, chemisorption	Wang et al. (2019a)
Magnetic γ-Fe_2_O_3_-GO-La	Fe coprecipitation, La impregnation, calcination	—	—	—	7.9	Ion exchange, complexation	Wen et al. (2015)
Zn-modifying slag	Impregnation	0.1 mm	58	—	7.9	Ion exchange	Sarkar et al. (2019)
ZrO_2_-graphene oxide	One-step ultrasound hydrothermal	—	632	—	7.3	Ligand exchange, electrostatic attraction	Mohan et al. (2016)
Hydrous Fe/Al GO	Coprecipitation, impregnation	200 μm	—	—	6	Electrostatic attraction, ion exchange	Kanrar et al. (2016)
Fe-modifying pumice	Impregnation	200 μm	25	—	3	—	Dehghani et al. (2016)
FeOOH–graphene oxide	In-suit hydrolysis	—	203	7.1	1.8	Ion exchange	Kuang et al. (2017)
Aluminum/olivine	Wet impregnation, calcination	—	—	—	—	Physical adsorption	Ghosal and Gupta (2018)
Polyhydroxy-iron	Impregnation	—	100	—	8	—	Muschin et al. (2021)
3D Y-GO hydrogels	GO-mixing SA, YCl_3_ cross-linking	—	147	15.26	6.74	Ion exchange	He et al. (2018)
Al2O3-chitosan biochar	HBO_3_ cross-linking, calcination	—	—	—	6	Ion exchange	Jiang et al. (2018)
Graphene oxide/eggshell	Impregnation	—	—	—	—	—	Nor et al. (2020)
Ce-SA/BC beads	SA/BC mixing, CeCl_3_ cross-linking, calcination	2 mm	237	3.97	8.26	Ion exchange, electrostatic attraction	Wei et al. (2022)
Ca-pectin-hydroxyapatite	Coprecipitation	—	157	3.1	7	Ion exchange, electrostatic attraction	Raghav et al. (2018)
Polypyrrole onto BC	Mixing, FeCl_3_ impregnation	—	—	—	8.6	Ion exchange	Wang et al. (2017c)

### 3.5 Modified Synthesis Methods

In summary of the aforementioned composite adsorbent synthesis methods (Tables 4, 5), it can be seen that the general principle of metal-modifying adsorbents is to coat the opposing surface with metal salts. The main methods of modification are chemical coprecipitation, impregnation, and hydrothermal methods, which are described here.

#### 3.5.1 Chemical Coprecipitation

**FIGURE 6:**
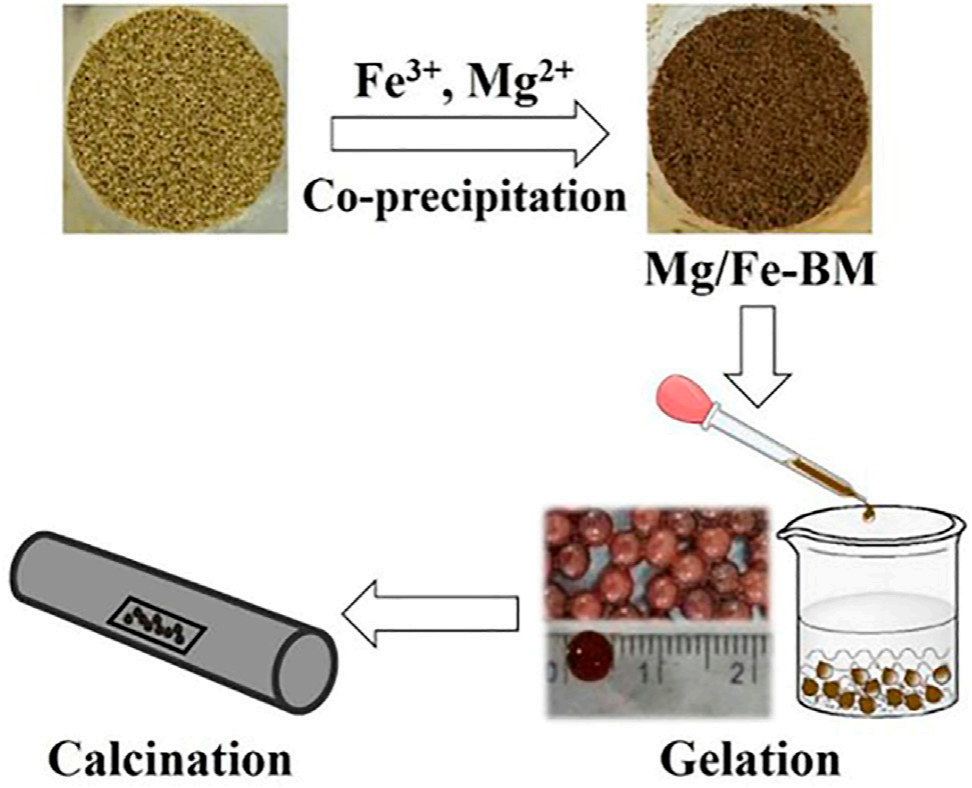
Coprecipitation method for preparation of MgFe_2_O_4_-doped biochar and ionic cross-linking process of composite sols (Wang et al., 2020).

Chemical coprecipitation is a common method for the preparation of multi-metals (Table 4) and metal-modified inorganic adsorbents (Table 5). In the preparation of multi-metal nanoparticles, the chemical coprecipitation method first mixes each metal salt solution in proportion to the atoms of the target product to be prepared to form an aqueous solution of the metal ions. The metal ions are then simultaneously precipitated out of the solution by the addition of appropriate precipitants to form hydroxide precipitate (Wu et al., 2013). The precipitates are separated out and then dried or calcined to obtain powdered multi-metal hydroxide/oxide nanoparticle adsorbents (Zhou et al., 2018). When modifying inorganic adsorbents with metals, the inorganic material is first put into a metal salt solution *via* methods such as adjusting the pH of the solution; the metal ions in the solution are induced to produce nano-metal particles that precipitate and load onto the surface and pore paths of the inorganic material, which are then dried or calcined to form composite adsorbents (Figure 6) (Wang et al., 2019b). The coprecipitation method is simple and not time-consuming. However, due to the different precipitation rates of different elements, there is sometimes a stratification of the precipitation, which makes the precipitate not uniformly dispersed and the composition of the product somewhat biased.

#### 3.5.2 Hydrothermal

**FIGURE 7:**
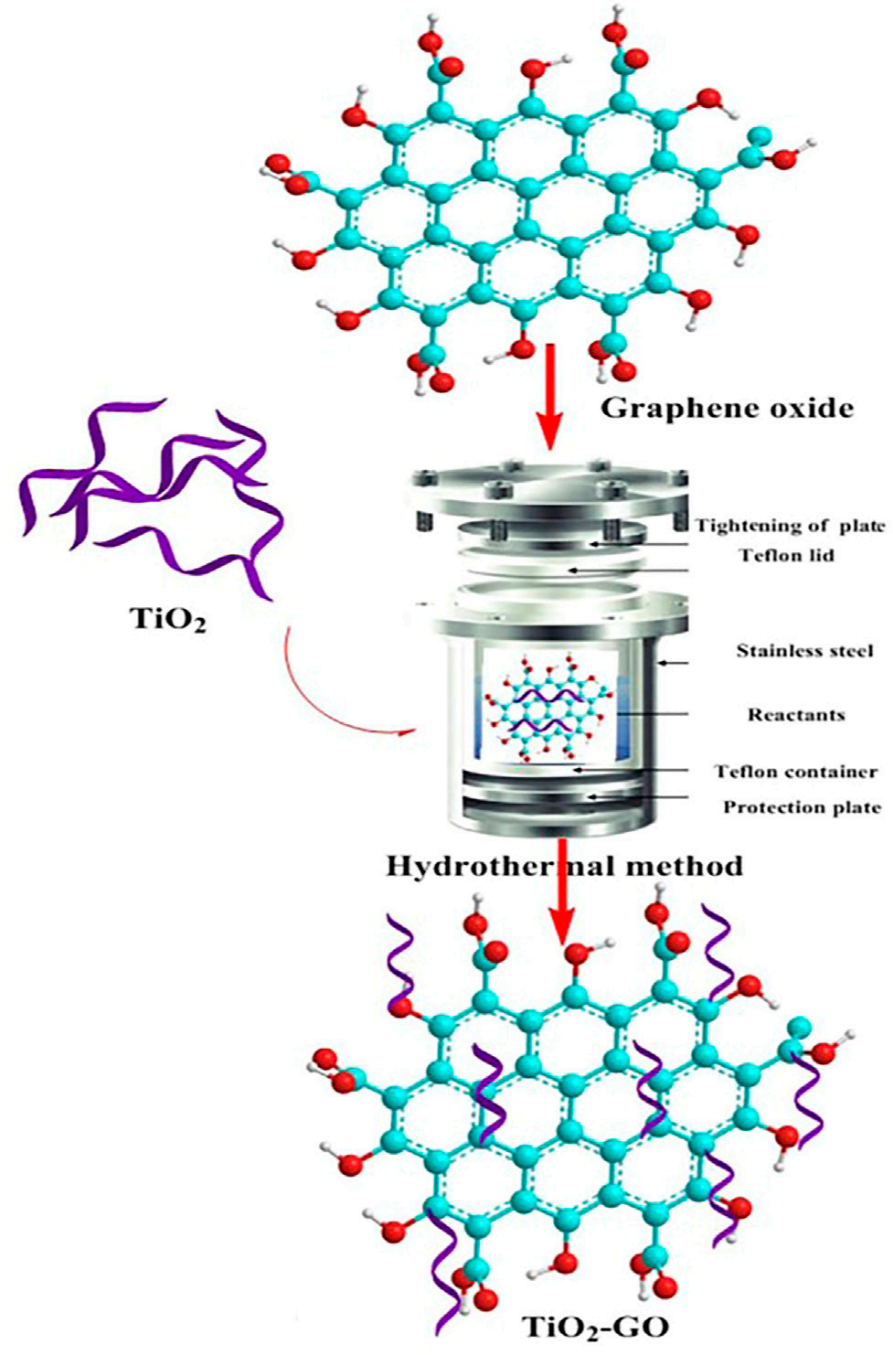
Hydrothermal for the preparation of TiO_2_-modified graphite (Nehra et al., 2019).

The hydrothermal method is also commonly used for the preparation of multi-metals (Table 4), and some studies have also been used for the modification of other materials by metals. Similarly, the metal ions are first prepared in a mixed solution in a certain proportion and then placed in a hydrothermal reactor at 423–573 K for a specific time. The principle of the hydrothermal method is that in a closed reaction environment, the precursor undergoes high temperature and pressure to fully dissolve in the solvent (Nehra et al., 2019). Then hydrolysis and nucleation according to a certain crystallization mode to grow nano-microcrystalline particles, to obtain a uniform particle size and good dispersion of composite powder (Figure 7). The nanomaterials prepared by the hydrothermal method have homogeneous morphology and the products are well dispersed. However, high pressure and temperature-resistant instrumentation are required, with long reaction times and production cycles, which are not conducive to mass production. Thus, it is often used in the laboratory to prepare nanomaterials with special morphologies for research. By controlling the crystallization time, crystallization temperature, and other factors, nanopowders with different morphologies can be prepared.

#### 3.5.3 Impregnation

**FIGURE 8:**
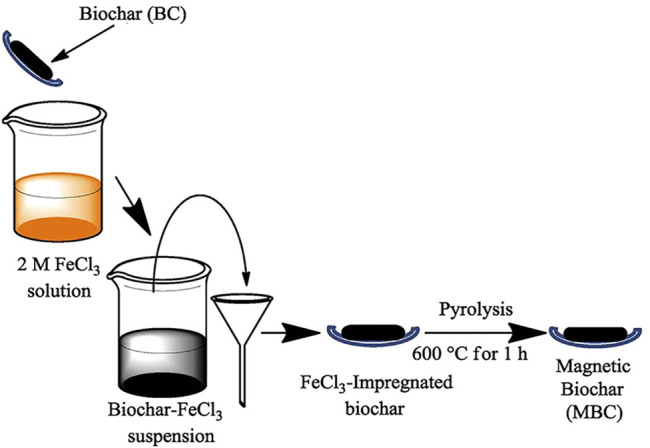
Preparation of magnetic biochar by impregnation and subsequent calcination (Bombuwala Dewage et al., 2018).

Impregnation is commonly used for metal-modified inorganic materials (carbon, clay, GO, etc. Table 5). The powdered inorganic material is first pre-treated by immersion in a metal salt solution (AlCl_3_, CaCl_2_, FeCl_3_, LaCl_3_, etc.). The metal ions in the solution can be loaded on an inorganic surface or internally *via* auxiliary heating and ultrasonic dispersion. The composite adsorbent is then dried or calcined (Figure 8). The impregnation preparation method is also facile but slightly more time-consuming, relying on the specific surface area of the inorganic material and bonding of the active sites on the surface to the metal.

Some preparations are calcined after coprecipitation, impregnation, or hydrothermal treatment (Figures 6–8). For example, metal-modified biochar is calcined to form metal oxide nanoparticles on the surface of BC, which further enhances the adsorption capacity (Table 5). LDH is also sometimes calcined (Table 4). During heating, LDH can be transformed into mixed metal oxides as the interlayer anions are eliminated by thermal decomposition. After the adsorbent is put into a fluoride solution, it will undergo a rehydration process. During rehydration, these oxides are in turn rebuilt into original layered structures by adsorbing various anions from the aqueous solution, known as the “memory effectˮ (Wu P. et al., 2017). The specific surface area and anion exchange capacity of LDH increases further after calcination. After coprecipitation or impregnation, the modification of biopolymers is generally achieved by the sol–gel method for the preparation of hydrogels (Wang A. et al., 2017).

### 3.6 Adsorption Mechanism

**FIGURE 9:**
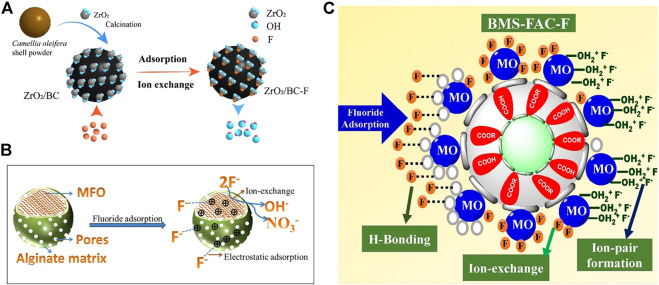
Respective adsorption mechanisms of composite adsorbents from different metal-modified materials. **(A)**: ion exchange (Mei et al., 2020), **(B)**: electrostatic attraction (Wu T. et al., 2017), **(C)**: ion pair (Raghav et al., 2019).

The adsorption mechanism can be divided into physical adsorption and chemisorption. Physical adsorption is generally considered to be caused by van der Waals forces, which are nonselective and reversible, and can be desorbed under certain conditions. Current research on the physical adsorption of fluoride ions is mainly based on electrostatic attraction and hydrogen bonding. Chemisorption is mainly the formation of chemical bonds between molecules and is described by the Langmuir model; the adsorption is selective and irreversible and desorption is more difficult. Physical adsorption depends mainly on the active pore volume and specific surface area (Wang H. et al., 2017), while chemisorption depends more on chemical or electro-affinity. The fluoride adsorption mechanism by various metal-modified adsorbents is summarized in Tables 4, 5. A total of four main adsorption mechanisms can be found: electrostatic attraction, ion exchange, hydrogen bonding, and complexation. The actual adsorption process is usually accompanied by several mechanisms (Figure 9).

When metal oxide enters the aqueous solution, the hydrogen ions are attracted to lone pair electrons of the oxygen element in metal oxide, forming a hydroxyl ligand (Zhang and Jia 2016). The fluoride removal by metal oxides and metal-modified composite adsorbents exploits the large number of hydroxyl groups on the surface (Figure 9A). When the solution pH is less than zero charge point (*pH*_*PZC*_) of the composite adsorbent, hydroxyl functional groups become protonated, forming OH_2_^+^ and are positively charged. The positive charge surface attracts negatively charged fluoride ions by electrostatic attraction (Figure 9B).

Fluoride is attracted to the surface of composite adsorbents for immobilization, but ion pairs (Figure 9C) are weakly interacting with each other and easily desorbed. Several studies have confirmed the involvement of hydroxyl groups in the adsorption reaction by FTIR and XPS characterization. F^−^ has the same charge and similar radius composition as OH^−^ and can replace OH^−^ in the structure of composite adsorbents (Figure 9A). F^−^ is bonded to a metal-occupying active site, OH^−^ is released, and the solution pH rises after adsorption. Most studies have been based on the anion exchange mechanism. As pH rises above the *pH*_*PZC*_ of the adsorbent, there is no significant decrease in adsorption. indicating that adsorption is mainly controlled by ion exchange. Generally, when the solution pH > 10, the large amount of free OH^−^ in the solution competes with F^−^, resulting in a significant decrease in adsorption capacity. Complexation between metals and fluoride has also been suggested (Suzuki et al., 2013).

Metal-modified composite adsorbents often have polar functional groups containing hydrogen, such as -OH, -COOH, and -NH_2_ (Yang et al., 2017). The shared electron pairs of polar functional groups are strongly biased toward oxygen or nitrogen, leaving the hydrogen atom almost naked. The lone pair electrons of electronegative fluoride will interact with the hydrogen atom forming a hydrogen bond with a bond angle of 180° and immobilize (Figure 9C).

## 4 Conclusion and Future Direction

Comparison of Table 2 with Tables 3, 6 reveals an overall increase in fluoride adsorption capacity of metal-modified composites. It indicates that most of the modifications are successful with application prospects. However, there are still many issues that need to be considered to achieve a big breakthrough in practical applications. The multi-metals enrich active sites for fluoride, but agglomeration and easy leaching are still problems, and individual preparation still requires some cost. Metal-modified biopolymers improve the stability of hydrogels, and metals can also be dispersed and immobilized in the macromolecular structure. However, it is reported that the dense surface of hydrogel makes it difficult for fluoride ions to enter the internal pores of beads, and beads sink easily so they have a limited contact area with fluoride. Metal-modified carbon, mineral clay, and other inorganic materials can also improve the dispersion and immobilization of metals to some extent, but there are still problems of dissolution, and loaded metals are easily dislodged and poorly recycled. Low-cost inorganic materials balance the price of rare earth metals and reduce the amount of metals, but at the same time, present the safety risk of waste use. The metal and inorganic materials are both in powder form, and the issue of separation and recycling has not been addressed. Studies combining metals, inorganic materials, and biopolymers appear to address the agglomeration and immobilization of metals, expanding the pore space and fluoride contact area of hydrogel beads, while improving the separation and recovery properties of inorganic materials. However, more than 90% of studies mentioned in this review avoided exploring metal dissolution concentrations and less than 10% of adsorbents were able to achieve more than 80% fluoride adsorption at the fifth cycle. Future studies will need to pay attention to the simplicity, efficiency, and cost of preparation procedure. Overall, the search for future defluoridation adsorbents is not limited to the requirement for increasing adsorption capacity. More important is the attention to cost levels, regeneration performance, separation and recovery, and safety issues for practical applications.

**TABLE 6:** Adsorption conditions and performance of fluoride by metal-modified carbon and other adsorbents.

Adsorbents	Adsorption condition					Isotherm model	Regeneration performance	*Q*_max_ (mg/g)	Ref
	Initial *C*_*F*_^*−*^ (mg/L)	Adsorbent dose (g/L)	Reaction pH	Temperature (K)	Equilibrium time (min)				
Wood biochar-La	40	5	6	298	50	Langmuir	53% at sixth cycle	164.20	Habibi et al. (2019)
Al-modified corn biochar	50	1	6.8	298	100	Langmuir	—	74.14	Zhang et al. (2021a)
MgO shell biochar	20	1	6–8	298	360	Langmuir	—	57	Wan et al. (2019)
Pomelo peel BC-La	10	2	6.5	298	1,200	Freundlich	66% at sixth cycle	19.86	Wang et al. (2018b)
ZrO_2_-seed shell biochar	10	1.6	3–9	298	180	Langmuir	50% at third cycle	9.63	Mei et al. (2020)
Magnetic biochar	10	2	2–9	308	5	Langmuir	—	9.04	Bombuwala Dewage et al. (2018)
Mg-Mn-Zr AC	10	1	2–10	303	180	Langmuir	—	26.27	Mullick and Neogi (2019)
Zr-impregnated AC	10	2	4	303	180	Langmuir	33% at fifth cycle	5.40	Mullick and Neogi (2018)
La-functionalized AC	200	1	7	303	180	Sips	—	10.51	Merodio-Morales et al. (2019)
Activated carbon@SnO_2_	10	0.3	6	303	180	Langmuir	80% at third cycle	4.60	Mohanta and Ahmaruzzaman (2018)
Ce-containing bone char	50	2	5	303	840	Sips	—	47.16	Zúñiga-Muro et al. (2017)
Magnetic bone biochar	20	5	8	298	1,440	Freundlich	38% at fourth cycle	5.23	Zhou et al. (2019a)
Graphene oxide with Ti	50	3.5	6	308	100	Langmuir	54% at sixth cycle	342	Nehra et al. (2019)
Al-polyacrylic acid	10	1	2	298	200	Freundlich	—	283.48	Xu et al. (2017)
CeO_2_@SiO_2_ microsphere	50	1.5	3	298	45	Langmuir	57% at fourth cycle	257.70	Wang et al. (2019a)
Magnetic γ-Fe_2_O_3_-GO-La	10	0.2	7	298	30	Langmuir	78% at sixth cycle	77.12	Wen et al. (2015)
Zn-modifying slag	50	0.5	5	298	30	Freundlich	—	60	Sarkar et al. (2019)
ZrO_2_-graphene oxide	25	0.5	7	303	50	Langmuir	59% at fifth cycle	45.7	Mohan et al. (2016)
Hydrous Fe/Al GO	10	3	5	308	60	Langmuir	—	22.9	Kanrar et al. (2016)
Fe-modifying pumice	3	0.7	3	298	50	Freundlich	—	21.74	Dehghani et al. (2016)
FeOOH–graphene oxide	25	2.5	2–10	298	120	Langmuir	—	17.672	Kuang et al. (2017)
Aluminum/olivine	10	2	6	303	60	Langmuir	—	12.94	Ghosal and Gupta (2018)
Polyhydroxy-iron	25	1	7	298	40	Freundlich	—	11.09	Muschin et al. (2021)
3D Y-based GO hydrogels	20	0.2	4	293	1,440	Langmuir	72% at third cycle	288.96	He et al. (2018)
Al2O3-chitosan biochar	20	1	3	298	1,440	Langmuir	—	196.1	Jiang et al. (2018)
Graphene oxide/eggshell	30	0.25	7	298	120	Langmuir	—	56.6	Nor et al. (2020)
Ce-SA/BC beads	10	1	3–9	293	20	Langmuir	—	34.86	Wei et al. (2022)
Ca-pectin-hydroxyapatite	10	1	7	298	30	Freundlich	—	28.47	Raghav et al. (2018)
Polypyrrole onto BC	10	1	6.5	298	—	Langmuir	53% at 4th cycle	18.52	Wang et al. (2017c)
